# A comparative study of the therapeutic efficacy of various intralesional immunotherapies in extragenital cutaneous warts

**DOI:** 10.3205/dgkh000611

**Published:** 2026-01-09

**Authors:** Nimisha Kabra, Rajesh Sinha, U.K Pallavi

**Affiliations:** 1 Department of Dermatology, Indira Gandhi Institute of Medical Sciences, Patna, Bihar, India

**Keywords:** extragenital cutaneous warts, Immunotherapy, MMR vaccine, BCG vaccine, vitamin D3 injection

## Abstract

**Background:**

Cutaneous warts are caused by human papilloma virus (HPV). Most of the current removal modalities are ablative which is associated with recurrence and scarring at the site. Immunotherapy can overcome these limitations, and also distant warts can be treated simultaneously.

**Aims:**

To compare the efficacy of three immunotherapeutic agents, measles-mumps-rubella (MMR) vaccine, Bacillus Calmette Guerin (BCG) vaccine, and vitamin D3 injection in the treatment of multiple extragenital cutaneous warts and to assess the safety and recurrence rates of different intralesional immunotherapeutic agents.

**Materials and methods:**

Sixty patients with extragenital cutaneous warts were enrolled in the study and randomized into three groups: Group A: MMR (0.5 mL of reconstituted MMR vaccine); Group B: BCG (0.1 ml BCG); and Group C: vitamin D3 (0.5 mL Inj. vitamin D3 600,000 IU; 15 mg/ml). A target wart was selected, and the intralesional injections were given at a three-week interval for a maximum of five doses. The response was observed in target and distant warts. Adverse effects were noted. Cases were followed up monthly for two months.

**Results:**

The baseline characteristics were comparable across MMR, BCG, and vitamin D3 groups. MMR showed significantly higher complete clearance at both injected (90%) and distant (80%) sites compared to BCG (60%, 40%) and vitamin D3 (25%, 20%) at the final follow-up. MMR was significantly superior to vitamin D3 (p=0.002) in injected warts and in distant warts (p=0.03) at last follow up. Intention-to-treat analysis and Kaplan-Meier survival confirmed a faster and more effective response with MMR (mean 5.3 weeks). Hazard ratios indicated a 95% and 99% lower probability of clearance with BCG and vitamin D3, respectively, compared to MMR. Pain was the most common adverse effect, being highest in vitamin D3 group (80%). There was recurrence in 3 cases in the MMR group, recurrence in 1 case and no recurrence in the vitamin D3 group upon follow-up.

**Conclusion:**

The intralesional MMR vaccine was found to be significantly more effective than BCG and vitamin D3 in treating extragenital cutaneous warts. This makes immunotherapy a promising modality for the treatment of multiple and recalcitrant extragenital cutaneous warts.

## Zusammenfassung

**Hintergrund:**

Hautwarzen werden durch das humane Papillomavirus (HPV) verursacht. Die meisten der derzeitigen Behandlungsmethoden sind ablativ, was mit einem Wiederauftreten und einer Narbenbildung an der Stelle verbunden ist. Mit der Immuntherapie lassen sich diese Einschränkungen überwinden und auch entfernte Warzen können gleichzeitig behandelt werden.

**Zielsetzung:**

Verglichen werden sollte die Wirksamkeit von drei immuntherapeutischen Mitteln, Masern-Mumps-Röteln-Impfstoff (MMR), Bacillus-Calmette-Guerin-Impfstoff (BCG) und Vitamin D3 zur Behandlung multipler extragenitaler kutaner Warzen einschließlich Bewertung der Sicherheit und der Rückfallrate.

**Material und Methoden:**

60 Patienten mit extragenitalen kutanen Warzen wurden in die Studie aufgenommen und in drei Gruppen randomisiert: Gruppe A: MMR (0,5 ml rekonstituierter MMR-Impfstoff), Gruppe B: BCG (0,1 ml BCG), und Gruppe C: Vitamin D3 (0,5 ml injiziertes Vitamin D3 600.000 IU; 15mg/ml). Die Zielwarze wurde ausgewählt, und die intraläsionalen Injektionen wurden in drei wöchentlichen Abständen für maximal fünf Dosen verabreicht. Reaktionen wurde bei der Zielwarze und bei entfernten Warzen beobachtet. Unerwünschte Wirkungen wurden festgestellt. Die Fälle wurden zwei Monate lang monatlich nachbeobachtet.

**Ergebnisse:**

Die Ausgangsdaten waren in den Gruppen MMR, BCG und Vitamin D3 vergleichbar. MMR zeigte eine signifikant höhere vollständige Clearance sowohl an den injizierten (90%) als auch an den entfernten Stellen (80%) im Vergleich zu BCG (60%, 40%) und Vitamin D3 (25%, 20%) bei der letzten Nachuntersuchung. MMR war bei injizierten Warzen (p=0,03) und bei entfernten Warzen (p=0,002) bei der letzten Nachuntersuchung dem Vitamin D3 signifikant überlegen. Die Intention-to-treat-Analyse und die Kaplan-Meier-Analyse bestätigten ein schnelleres und wirksameres Ansprechen auf MMR (durchschnittlich 5,3 Wochen). Die Hazard Ratios zeigten eine um 95 % bzw. 99% geringere Wahrscheinlichkeit einer Clearance bei BCG und Vitamin D3 im Vergleich zu MMR. Schmerzen waren die häufigste unerwünschte Wirkung, am häufigsten in der Vitamin-D3-Gruppe (80%). In der MMR-Gruppe kam es in 3 Fällen zu einem Rezidiv, in der Vitamin-D3-Gruppe in 1 Fall zu einem Rezidiv und in der Nachbeobachtung zu keinem Rezidiv.

**Schlussfolgerung:**

Intralesionaler MMR-Impfstoff erwies sich bei der Behandlung extragenitaler kutaner Warzen als deutlich wirksamer als BCG und Vitamin D3. Das macht die Immuntherapie zu einer vielversprechenden Methode für die Behandlung multipler und rezidivierender extragenitaler kutaner Warzen.

## Introduction

Human papillomavirus (HPV) is a small, non-enveloped, double-stranded DNA virus with a preference for epithelial tissues and is known to cause papillomas or warts. It infects both keratinized and non-keratinized epithelial surfaces, leading to the development of cutaneous, genital, oral, and laryngeal warts. The virus typically gains entry through breaches in the epithelium and targets the basal cell layer [1]. Autoinoculation, where the virus spreads from an existing wart to adjacent healthy skin, is commonly seen, particularly in flat and digital warts. Studies using polymerase chain reaction (PCR) techniques have detected HPV DNA not only in the skin near HPV-related lesions but also in the skin of healthy individuals [2]. These findings help explain us the frequent recurrence of warts

Multiple therapeutic options exist for managing warts, including physical modalities such as cryotherapy, electrosurgery, ablative lasers, and surgical excision; chemical agents such as salicylic acid and trichloroacetic acid; as well as anti-proliferative drugs, e.g., podophyllin, 5-fluorouracil, and bleomycin. However, none of these approaches have demonstrated complete and consistent effectiveness in eradicating the condition [3].

Due to drawbacks associated with conventional treatments such as scarring and high recurrence rates, immunotherapy has gained increasing popularity, particularly for managing stubborn, recurrent, and widespread warts, as well as lesions in challenging areas, e.g., the periungual and palmoplantar regions. Immunotherapy refers to a form of biological treatment that enhances or modulates the immune system to aid the body in combating infections, cancer, and various diseases. These agents can be delivered via topical application, intralesional injection, or systemic administration [4]. Systemic immunotherapeutic options include agents such as interferons and contact sensitizers, while intralesional immunotherapy utilizes antigens such as *Candida albicans*, the measles-mumps-rubella (MMR) vaccine, Trichophyton, and tuberculin-based antigens like purified protein derivative (PPD), Mycobacterium w vaccine, and Bacillus Calmette-Guérin (BCG) [5].

Intralesional immunotherapy functions by activating the immune system to induce a delayed-type hypersensitivity response targeting both the introduced antigens and the wart tissue. This immune reaction prompts the release of Th1 cytokines, which stimulate cytotoxic T cells and natural killer cells to combat the HPV infection. In contrast to traditional therapies, this method has the advantage of clearing not only the directly treated warts but also distant, untreated lesions [6]. The operational mechanism of MMR vaccine, BCG vaccine and vitamin D3 is based on the notion of immunotherapy. Vitamin D3 regulates cell growth and differentiation while also exerting immunomodulatory effects. Its action is mediated through the vitamin D receptor (VDR), which is found in skin cells such as keratinocytes, melanocytes, fibroblasts, and various immune cells. Activation of VDR promotes the expression of antimicrobial peptides, enhancing the skin’s immune defence [7]. The BCG vaccine is believed to exert its effects by activating macrophages, T lymphocytes, and natural killer cells. Additionally, Toll-like receptor 7 (TLR7) may contribute to its mechanism of action [8].

Considering the relatively safe profile of immunotherapy for treating warts and previous studies reporting high rates of wart resolution, we designed the current study to assess and compare the effectiveness of intralesional injections of the MMR vaccine, BCG vaccine, and vitamin D3 injection in managing extragenital cutaneous warts. To the best of our knowledge, this is the first study to directly compare the therapeutic outcomes of these specific immunotherapeutic agents.

## Materials and methods

Study design: The study was conducted as a double-blind, randomized, parallel-group, active-controlled study. Ethical approval was secured from the Institutional Ethics Committee prior to commencement (05-10-2023; No. 1280/IEC/IGIMS/2023), and written informed consent was obtained from all participants. 

The study included patients aged between 12 and 65 years, who presented to the dermatology outpatient department, with clinically diagnosed extragenital cutaneous warts. Eligibility criteria required participants to have more than two warts and no history of wart treatment within the past four weeks. The study excluded pregnant or breastfeeding women, individuals with immunosuppression due to underlying diseases or medications, patients with mucosal warts, those who did not consent, individuals with severe organ dysfunction, those unable to attend monthly follow-ups, and users of alcohol or other substances. Enrolment was carried out according to the defined inclusion and exclusion criteria, with informed written consent obtained from all participants. Each patient underwent a comprehensive clinical evaluation, along with baseline investigations including a complete blood count, fasting blood sugar, serum urea and creatinine, liver function tests, and HIV screening.

The study included 60 patients, who were further randomly distributed into 3 groups by computer generated randomization list: Group A-MMR group, Group B- BCG group, Group C- vitamin D3 group. Freeze dried MMR vaccine single-use vials stored at 2°C–8°C was reconstituted with 0.5 mL of provided diluent (distilled water) and was given intralesionally up to 0.5 mL into a target antigen. Group B received 0.1 ml BCG Vaccine IP (freeze dried) which was reconstituted with 1 ml sodium chloride injection IP (0.9%) into a target antigen. Group C patients received a maximum of 0.5 mL Inj. vitamin D3 (600,000 IU; 15mg/ml) in each session after injection of intralesional lignocaine. Injections were given using a 27 G insulin syringe. The session was repeated at 3 weekly intervals for a maximum of 5 sessions or until complete resolution of warts, whichever was earlier. Patients were followed monthly for two months to evaluate for any recurrences. All adverse events were recorded. The target wart was defined as the largest wart into which immunogen was injected. In cases where the size of the target wart decreased significantly between the sessions, the largest wart from remaining lesions was considered as the target wart for that session. A distant site was defined arbitrarily as un injected wart that is away from the target wart.

### Evaluation of response

Patient and physician global assessment using a visual analog scale score and photographic comparison were used to assess decrease in size and number of warts and thus the response to treatment (Table 1 (Tab. 1)). 

### Statistical analysis

Categorical variables were expressed as frequency and percentage and analysed using Chi square test. Statistical significance was set at p<0.05. Continuous variables were expressed as mean and standard deviation and analysed using ANOVA. Kaplan-Meier analysis was done for time to clearance. The Cox proportional hazard model was used to calculate the hazard ratio. All statistical analysis was done using Epi info version 7.2.1.0 and JAMOVI version 4.0 statistical software. 

## Results

The baseline demographic, morphological, and clinical characteristics of the study participants across three treatment groups MMR (Group A), BCG (Group B), and vitamin D3 (Group C) are depicted in Table 2 (Tab. 2). The mean age across the groups was comparable, with no significant difference (p=0.715). The average duration of warts was slightly longer in the BCG group (7.6±3.65 months) compared to the MMR (5.25±2.86 months) and vitamin D3 (6.8±4.15 months) groups, but this difference was not statistically significant (p=0.119). The distribution of wart types such as verruca vulgaris, verruca plana, and palmoplantar did not vary statistically significantly (p=0.696). Likewise, the history of past or familial occurrence of warts and the anatomical site of lesions (face and neck, trunk and extremities, palms and soles) had p-values above 0.05.

At the 3^rd^ week, the MMR group showed a markedly higher complete response rate (35%) compared to BCG and vitamin D3 groups (both 5%), with the difference being statistically significant (p<0.001) in the injected warts. MMR demonstrated a markedly superior response compared to both BCG (p=0.005) and vitamin D3 (p=0.001) (Table 3 (Tab. 3)). By the final follow-up (15^th^ week), the MMR group again showed the highest complete clearance rate at 90%, followed by BCG at 60%, and vitamin D3 at only 25% (p=0.006) with the difference being statistically significant. MMR continued to show significantly better outcomes than vitamin D3 (p=0.002), while the difference between MMR and BCG (p=0.070) and between BCG and vitamin D3 (p=0.206) was not statistically significant.

**Figure 1:**
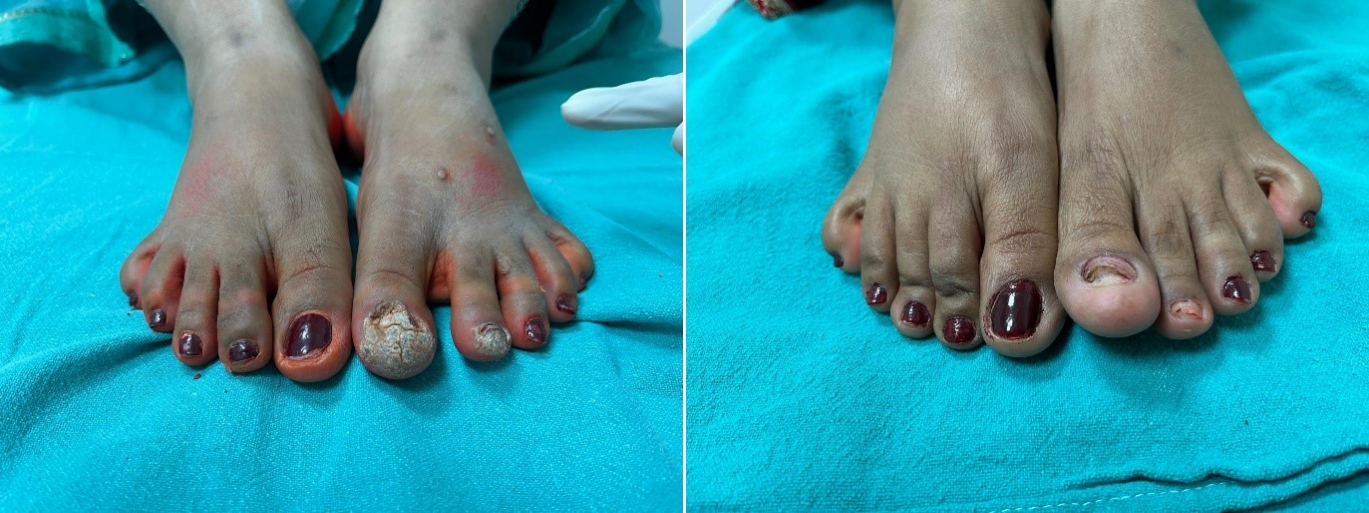
Left: warts treated with intralesional MMR at week 0 (baseline), Right: patient at 15^th^ week (last follow-up) with complete response.

**Figure 2:**
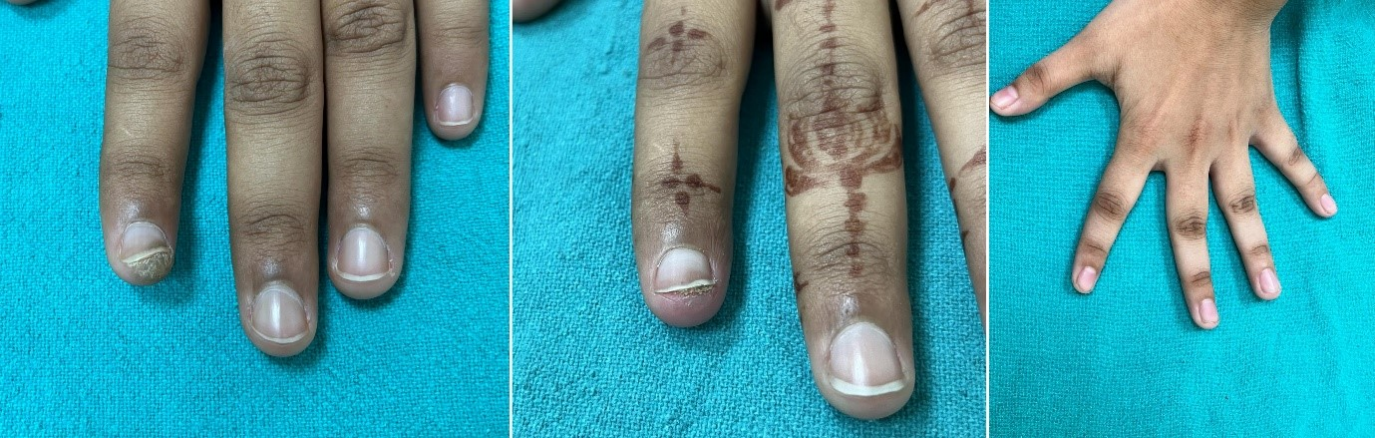
Left: warts treated with intralesional BCG at week 0 (baseline); middle: warts at 9^th^ week (excellent resolution); right: complete resolution of warts at 15^th^ week.

**Figure 3:**
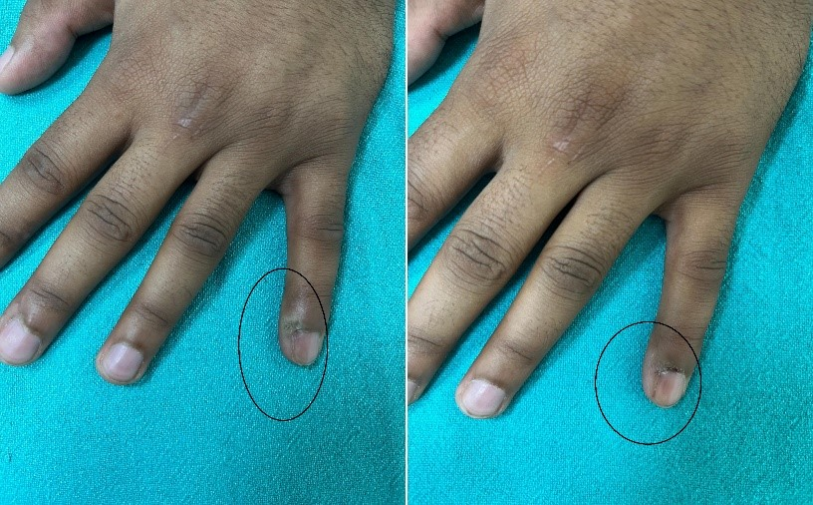
Left: warts treated with intralesional injection vitamin D3 at week 0 (baseline); right: complete resolution of warts at 15^th^ week.

Table 4 (Tab. 4) assesses the response of distant (uninjected) warts across the three groups. At the 3^rd^ week, the MMR group showed superior efficacy, with 30% achieving complete response compared to just 5% each in the BCG and vitamin D3 groups (p=0.003). By the final follow-up (15^th^ week), complete clearance of distant warts was seen in 80% of MMR patients, 40% of BCG patients, and only 20% of vitamin D3 patients (p=0.016) (Figure 1 (Fig. 1), Figure 2 (Fig. 2), Figure 3 (Fig. 3)). MMR had significantly better distant wart clearance than both BCG (p=0.020) and vitamin D3 (p=0.003) at the 3^rd^ week. The response between BCG and vitamin D3 was not significant (p=0.630). By the 15^th^ week, MMR maintained significantly superior efficacy over vitamin D3 (p=0.003). However, differences between MMR vs BCG (p=0.105) and BCG vs vitamin D3 (p=0.718) were not statistically significant.

**Figure 4:**
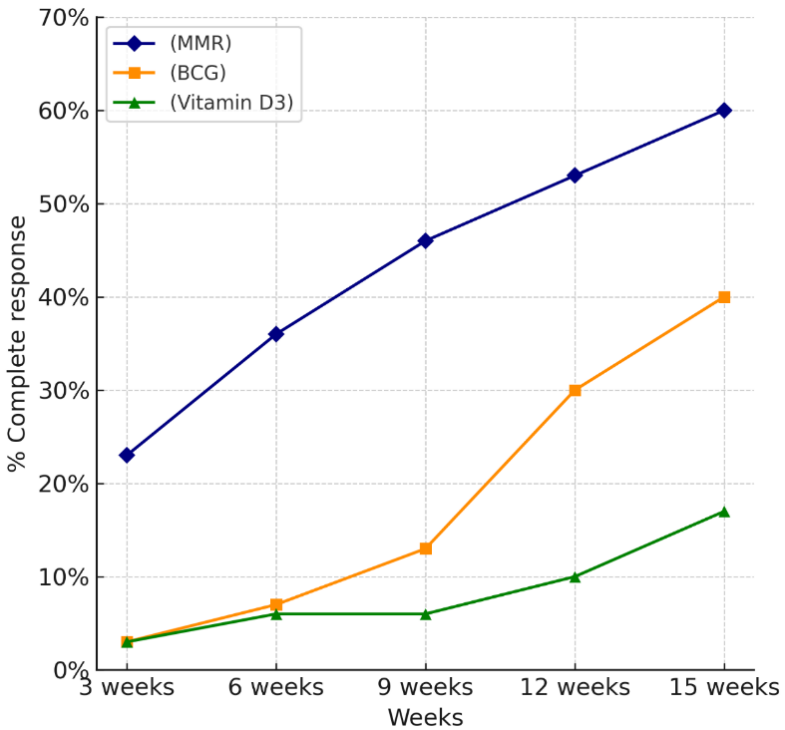
Complete response rate at injected site at different follow-up times (ITT analysis) (curves)

According to intention-to-treat analysis (ITT), at the injected site, 60% of MMR patients achieved complete response by the 15^th^ week, significantly higher than BCG (40%) and vitamin D3 (16.7%) (p=0.003) (Table 5 (Tab. 5), Figure 4 (Fig. 4)).

**Figure 5:**
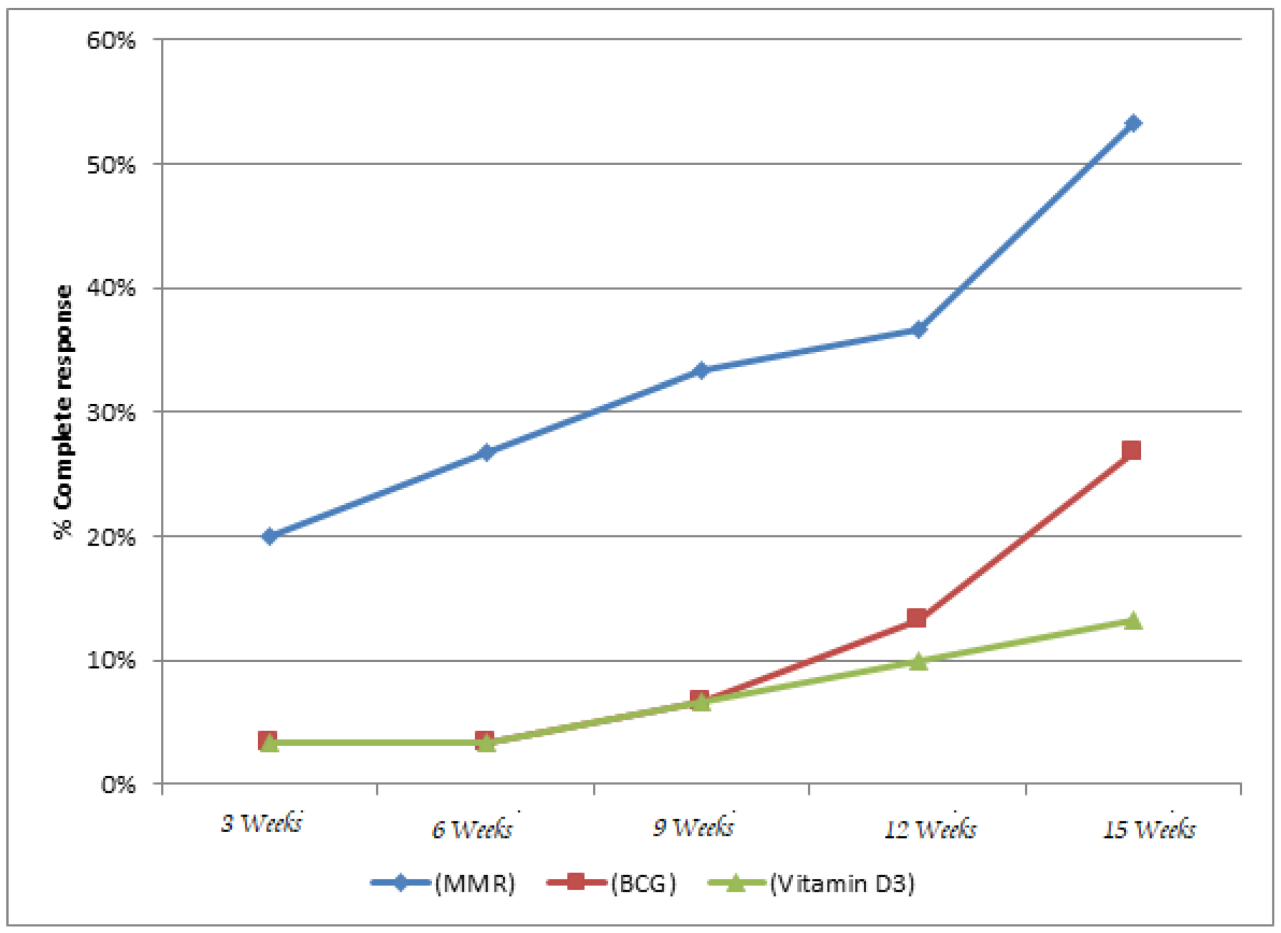
Complete response rate at distant site at different follow-up times (ITT analysis)

Similarly, at distant sites, the MMR group showed 53.3% complete clearance, compared to 26.7% in the BCG group and 13.3% in the vitamin D3 group, also with a statistically significant difference (p=0.003) (Table 6 (Tab. 6), Figure 5 (Fig. 5)).

**Figure 6:**
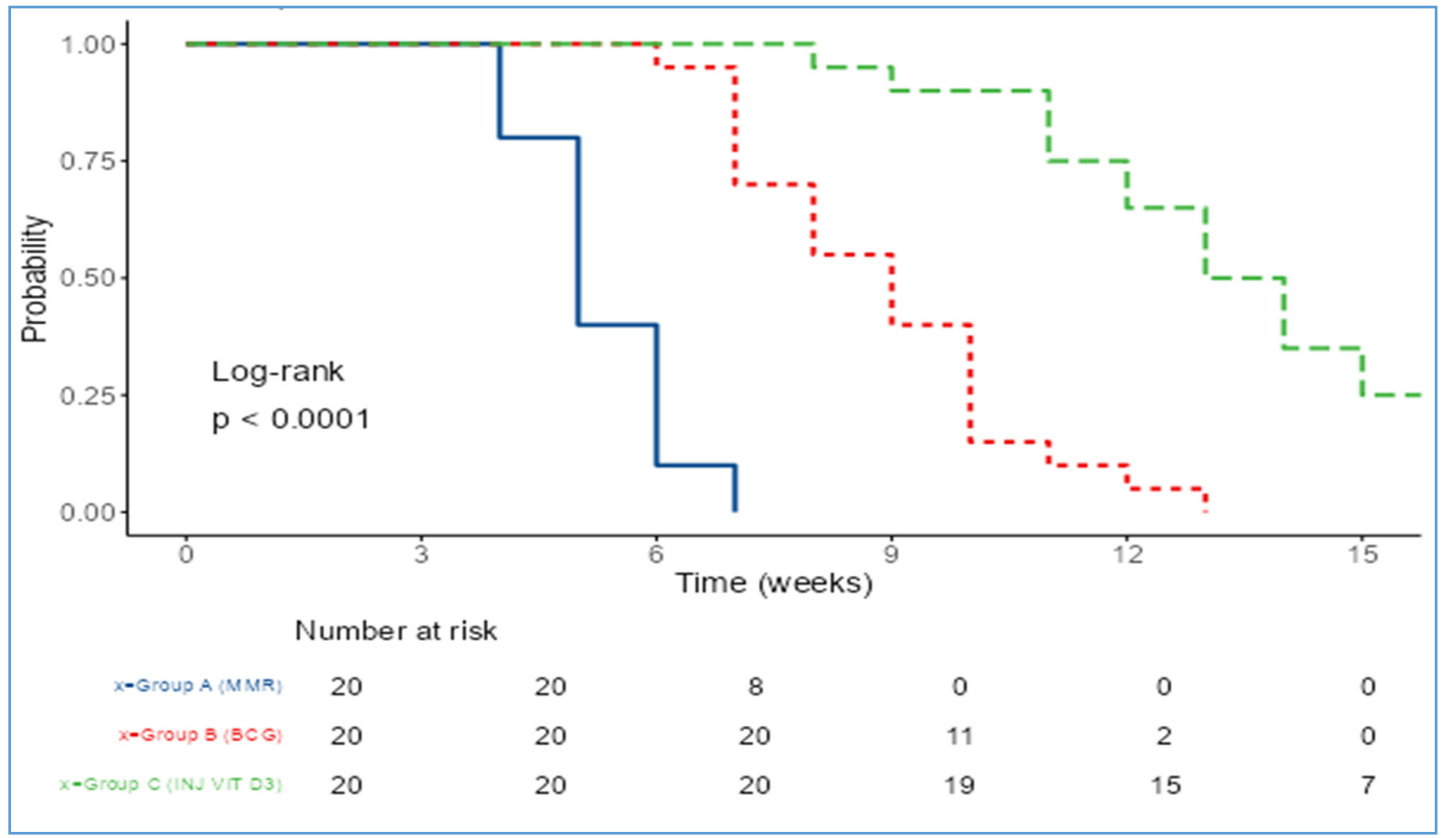
Kaplan Meier analysis for time to resolution

The Kaplan-Meier analysis (Figure 6 (Fig. 6)) for time to resolution of warts demonstrated that the MMR group had the fastest and most favorable treatment outcome among the three groups. 

The mean time to complete clearance was 5.3±0.92 weeks for MMR, significantly shorter than 8.9±1.86 weeks for BCG and 13.35±2.54 weeks for vitamin D3, with a highly significant p-value (p<0.001) (Table 7 (Tab. 7)). 

The median time to clearance further supports this trend, being 5 weeks for MMR, 9 weeks for BCG, and 13.5 weeks for vitamin D3 (Table 8 (Tab. 8)).

Using the MMR group as the reference, the Cox proportional hazards analysis showed that the BCG group had a hazard ratio (HR) of 0.05 (95% CI: 0.02–0.15, p<0.001). The vitamin D3 group had an even lower HR of 0.01 (95% CI: 0.00–0.03, p<0.001), reflecting a 99% reduced probability of clearance relative to MMR (Table 9 (Tab. 9)). 

Pain was the most commonly reported side effect, with a significantly higher incidence in the vitamin D3 group (80%) compared to BCG (35%) and MMR (20%) (p<0.001). Intergroup comparisons revealed that pain was significantly more frequent in vitamin D3 recipients than in both MMR (p<0.001) and BCG groups (p=0.011), while the difference between MMR and BCG was not significant (p=0.479). Other adverse effects, e.g., erythema, swelling, fever, and nodularity, were infrequent and statistically non-significant across groups. There was recurrence of 3 cases in MMR group, 1 case in BCG and no case recurred in vitamin D3 group.

## Discussion

Although warts may resolve on their own within 1 to 2 years, they can sometimes persist for extended periods, leading to physical discomfort, emotional distress, and a negative impact on the patient's quality of life. Recurrence with the appearance of new lesions can happen when the immune system is unable to effectively recognize and eliminate the HPV infection [9]. The standard approach for wart treatment typically involves local destruction of the affected tissue. However, this method often leads to a high risk of recurrence and potential scarring. Moreover, it is not ideal for treating warts located on multiple sites or in sensitive areas such as the face, palms, or soles. Immunotherapy through intralesional injections is believed to function by triggering a systemic T-cell mediated immune response. This stimulates the release of Th1 cytokines, including interleukin-2 and interferon-gamma. Additionally, delivering the treatment directly into the lesion may help enhance the localized immune reaction [5].

Immunotherapy is a form of biological treatment that involves using specific agents to either boost or suppress the immune system, aiding the body in combating infections, cancer, and other diseases. It can be categorized into activation immunotherapy, which stimulates or enhances immune responses (commonly used in cancers and infections), and suppression immunotherapy, which reduces immune activity (typically used for autoimmune disorders). While its application is well-recognized in the treatment of malignancies, immunotherapy is increasingly being utilized in the management of infectious conditions as well [10]. Substances used for intradermal or intralesional immunotherapy encompass a variety of biological agents, including protein extracts such as tuberculin, bacterial preparations like Bacillus Calmette-Guérin (BCG) and Mycobacterium w vaccine, fungal components such as *Candida albicans* and *Trichophyton*, and viral agents including the measles, mumps, and rubella (MMR) vaccine, as well as autoinoculated wart tissue [11], [12]. Previous studies involving MMR, BCG and vitamin D3 as immunotherapies in cutaneous warts have been summarized in Table 10 (Tab. 10). The findings of this study may represent one of the earliest randomized comparisons involving three immunotherapeutic agents MMR, BCG, and vitamin D3.

This study aimed to evaluate the efficacy and safety, as well as adverse effects of various modalities of intralesional therapy for the treatment of multiple cutaneous extragenital warts with the determination of recurrence rates at follow-up. In our study, 60 patients were selected based on the predefined inclusion and exclusion criteria. Our findings indicate that MMR was the most efficacious among the three, demonstrating complete clearance at the injected site in 90% and at distant sites in 80% of patients by the end of 15 weeks (Figure 1 (Fig. 1)). BCG followed with 60% and 40% clearance at local and distant sites (Figure 2 (Fig. 2)), respectively, while vitamin D3 showed the least efficacy with 25% and 20% clearance (Figure 3 (Fig. 3)).

Chauhan et al. [13] reported an 82.4% complete response with MMR, closely aligning with our 90% clearance. Similarly, Nofal et al. [6] and Chandran et al. [14] observed MMR effectiveness at 81.9% and 63%, respectively. The variation across studies could be due to differences in sample size, vaccine dose, number of treatment sessions, and follow-up duration. Our use of a higher dose (0.5 mL) and three-week intervals for up to five sessions may have contributed to the enhanced response compared to studies with lower MMR volumes or fewer sessions. 

In relation to BCG, our findings of 60% clearance at the local site and 40% at distant sites are comparable to studies by Jaisinghi et al. [15] (75.5%) and Rao and Haqqani [16] (70%). Srinivasa et al. [17] even reported a 90% clearance, which might reflect differences in wart types or host immune response. Ebrahim et al. [18] also demonstrated a 63.8% efficacy with BCG. In a comparative study by Shaker et al. [19], BCG showed the highest efficacy (70%) compared to MMR and tuberculin, reinforcing its role as a reliable option. The time to clearance with BCG in our study averaged 8.9 weeks, and while slightly slower than MMR

Vitamin D3 showed the least efficacy in our cohort, achieving only 25% and 20% complete clearance at the local and distant sites, respectively. These findings contrast with those of Al-Sabak et al. [20]. and Raghukumar et al. [21], who reported higher success rates of 81.9% and 90%. The variation could be attributed to differences in dosage (we used 0.5 mL of 600,000 IU), technique, or patient profiles. Notably, although Singh [22] found vitamin D3 to be slightly more effective than BCG (42.86% vs. 37.5%), our study clearly demonstrated better results with BCG. Vitamin D3 also had the slowest time to clearance (mean 13.35 weeks) and the highest incidence of pain (80%), making it less favorable in terms of patient tolerance. When comparing the intention-to-treat (ITT) and Kaplan-Meier analysis findings from our study with those of Lahoria et al. [23], significant differences in treatment outcomes are evident. Our ITT analysis showed MMR achieving complete clearance in 60% of patients at the injected site and 53.3% at distant sites, with a mean time to clearance of 5.3±0.92 weeks. In contrast, Lahoria et al. [23] reported lower ITT efficacy for MMR at 55% (injected) and 53% (distant) with a longer mean clearance time of 7.3 weeks and no statistically significant difference between treatment arms (p>0.05). Moreover, our Kaplan-Meier survival analysis demonstrated a significantly faster response with MMR compared to BCG and vitamin D3 (p<0.001), while the survival analysis by Lahoria et al. found no significant differences between MMR, MIP, vitamin D3, and even placebo (p=0.736). In our study, MMR demonstrated the highest efficacy in terms of both local and distant wart clearance, as well as the shortest time to complete resolution (mean: 5.3±0.92 weeks). Given MMR’s consistently superior therapeutic performance across multiple outcome measures and because it is the most extensively studied therapy [24], MMR was selected as the reference group in the Cox proportional hazards analysis. The hazard ratio for BCG was 0.05 and 0.01 for vitamin D3, both with highly significant p-values (p<0.001). This means that, compared to MMR, patients treated with BCG were 95% less likely and those with vitamin D3 were 99% less likely to achieve complete wart clearance at any given time. These results emphasize that MMR is not only the most effective in achieving faster and more complete resolution of warts, but also significantly outperforms BCG and vitamin D3 in terms of treatment success over time. All three treatments were generally safe, with most side effects being mild and self-limited. Pain was significantly more common with vitamin D3 (80%) compared to BCG (35%) and MMR (20%). Other side effects, e.g., erythema, swelling, and fever, were infrequent and comparable across groups. Overall, MMR had the most favorable safety profile, while vitamin D3 was associated with the highest discomfort.

## Conclusion

This study demonstrates that the intralesional MMR vaccine is significantly more effective than BCG and vitamin D3 in treating extragenital cutaneous warts, offering faster and more sustained clearance at both local and distant sites. BCG showed moderate efficacy, while vitamin D3 had the least therapeutic response and higher incidence of pain. MMR also had the highest hazard ratio for clearance, highlighting its superior clinical utility. Thus, MMR emerges as a safe, well-tolerated, and highly effective immunotherapeutic option for the management of multiple cutaneous warts.

## Notes

### Ethical approval 

Ethical approval was given by the Institutional Ethics Committee (Letter no.: 05-10-2023; No. 1280/IEC/IGIMS/2023)

### Funding

None. 

### Competing interests

The authors declare that they have no competing interests.

## Figures and Tables

**Table 1:**
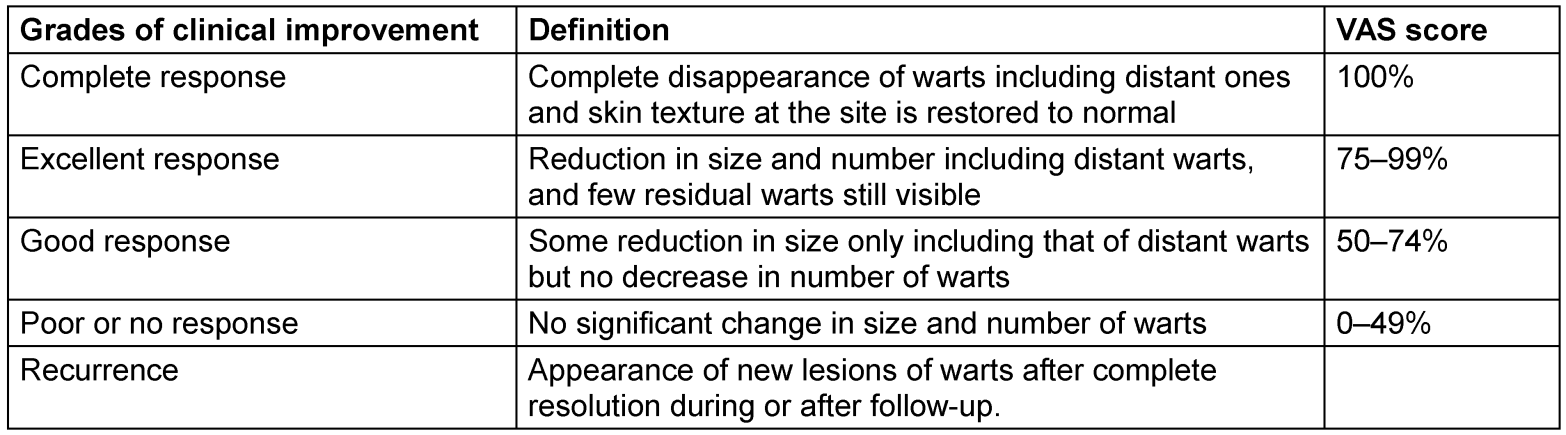
Visual analog scale score

**Table 2:**
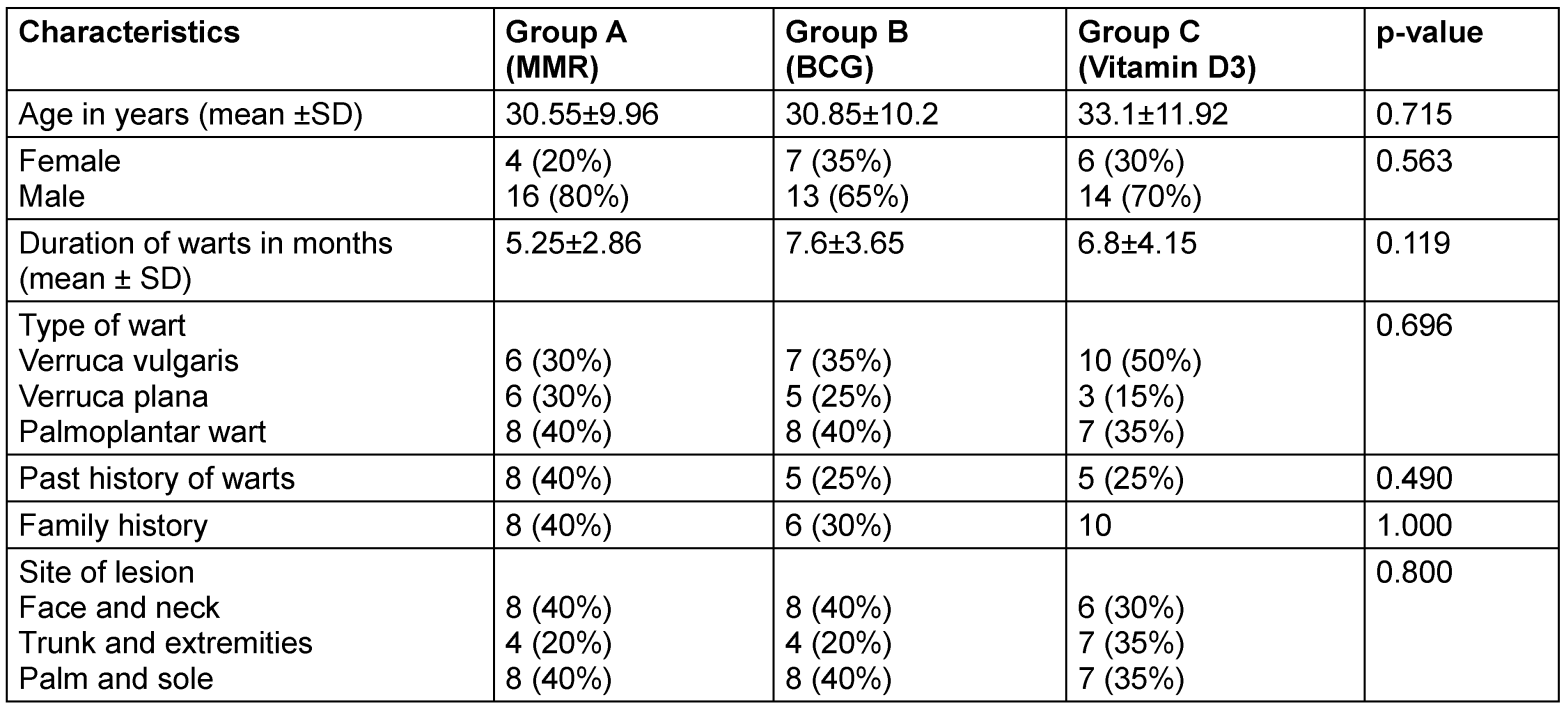
Demographic data, morphology and site of involvement

**Table 3:**
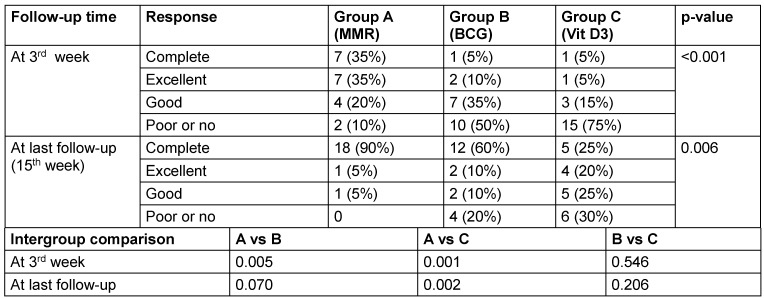
Comparison of response at different follow-up times in injected warts

**Table 4:**
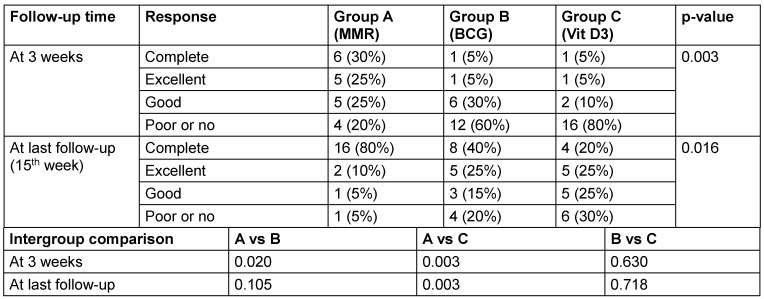
Comparison of response at different follow up times in distant warts

**Table 5:**
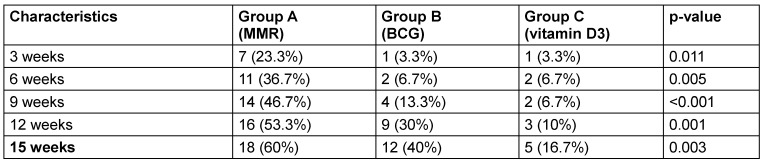
Complete response rate at injected site at different follow-up times (ITT analysis)

**Table 6:**
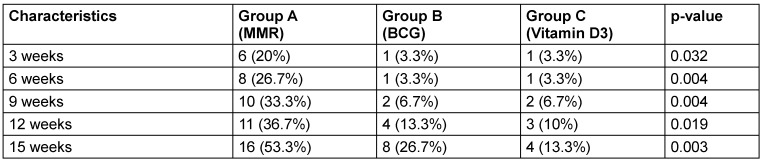
Complete response rate at distant site at different follow up time (ITT analysis)

**Table 7:**
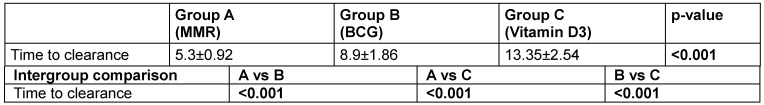
Comparison of time to clearance (weeks) among study groups

**Table 8:**
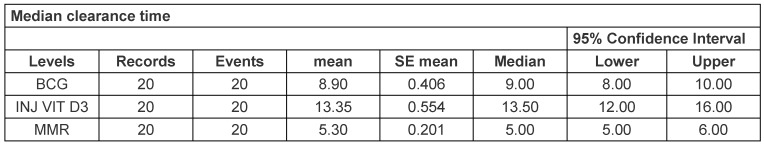
Median clearance time

**Table 9:**
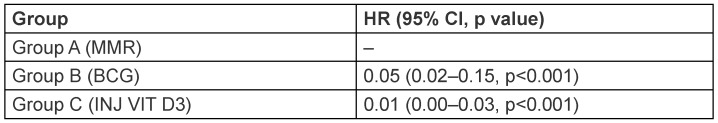
Cox analysis

**Table 10:**
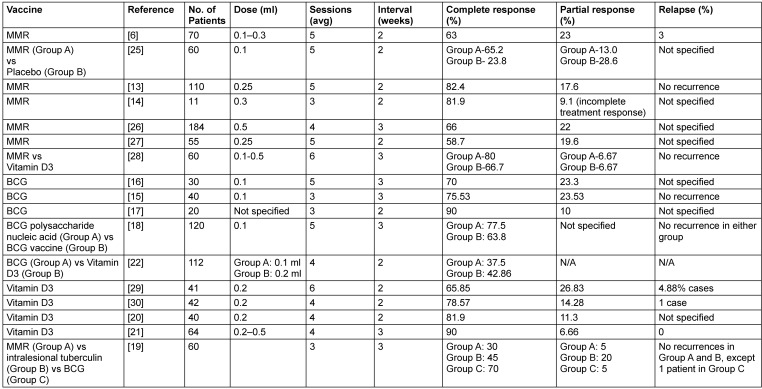
Clinical studies on intralesional immunotherapy for cutaneous warts
